# High-Risk Features Are Prognostic in dMMR/MSI-H Stage II Colon Cancer

**DOI:** 10.3389/fonc.2021.755113

**Published:** 2021-10-25

**Authors:** Amr Mohamed, Renjian Jiang, Philip A. Philip, Maria Diab, Madhusmita Behera, Christina Wu, Olatunji Alese, Walid L. Shaib, Tyra M. Gaines, Glen G. Balch, Bassel F. El-Rayes, Mehmet Akce

**Affiliations:** ^1^ Case Comprehensive Cancer Center, Case Western Reserve University, Cleveland, OH, United States; ^2^ Winship Research Informatics Shared Resource, Winship Cancer Institute, Emory University, Atlanta, GA, United States; ^3^ Karmanos Cancer Institute, Wayne State University, Detroit, MI, United States; ^4^ Department of Hematology and Medical Oncology, Winship Cancer Institute, Emory University School of Medicine, Atlanta, GA, United States; ^5^ Division of Colorectal Surgery, Department of Surgery, Emory University School of Medicine, Atlanta, GA, United States

**Keywords:** high risk, stage II, adjuvant chemotherapy, colon, cancer

## Abstract

**Background:**

High-risk features, such as T4 disease, bowel obstruction, poorly/undifferentiated histology, lymphovascular, perineural invasion, and <12 lymph nodes sampled, indicate poor prognosis and define high-risk stage II disease in proficient mismatch repair stage II colon cancer (CC). The prognostic role of high-risk features in dMMR/MSI-H stage II CC is unknown. Similarly, the role of adjuvant therapy in high-risk stage II CC with dMMR/MSI-H (≥1 high-risk feature) has not been studied in prospective trials. The aim of this analysis of the National Cancer Database is to evaluate the prognostic value of high-risk features in stage II dMMR/MSI-H CC.

**Methods:**

Univariate (UVA) and multivariate (MVA) Cox proportional hazards (Cox-PH) models were built to assess the association between clinical and demographic characteristics and overall survival. Kaplan–Meier survival curves were generated with log-rank tests to evaluate the association between adjuvant chemotherapy in high-risk and low-risk cohorts separately.

**Results:**

A total of 2,293 stage II CC patients have dMMR/MSI-H; of those, 29.5% (*n* = 676) had high-risk features. The high-risk dMMR/MSI-H patients had worse overall survival [5-year survival and 95%CI, 73.2% (67.3–78.1%) *vs*. 80.3% (76.7–83.5%), *p* = 0.0001]. In patients with stage II dMMR/MSI-H CC, the high-risk features were associated with shorter overall survival (OS) along with male sex, positive carcinoembryonic antigen, Charlson–Deyo score >1, and older age. Adjuvant chemotherapy administration was associated with better OS, regardless of the high-risk features in dMMR/MSI-H (log-rank test, *p* = 0.001) or not (*p* = 0.0006). When stratified by age, the benefit of chemotherapy was evident only in patients age ≥65 with high-risk features.

**Conclusion:**

High-risk features are prognostic in the setting of dMMR/MSI-H stage II CC. Adjuvant chemotherapy may improve survival specifically in patients ≥65 years and with high-risk features.

## Introduction

Colorectal cancer is the third most common cancer and the second leading cause of cancer-related mortality in the United States. It is estimated that 104,270 new cases of colon cancer (CC) will be diagnosed in 2021 in the US (1). Approximately 28% of patients with CC have stage II disease at presentation (2). The risk stratification of patients with stage II CC is dependent on molecular and clinicopathologic features. A prognostic role of high-risk features, such as T4 disease, bowel obstruction, poorly/undifferentiated histology, lymphovascular, perineural invasion, and <12 lymph nodes sampled, is well established, and high-risk features increase the risk of cancer recurrence and the benefit from adjuvant therapy in patients with microsatellite stable disease stage II CC (3–5). A subgroup of high-risk patients with stage II CC with T4 disease may have a statistically inferior survival compared to those with stage IIIa tumors (6). Adjuvant chemotherapy improves progression-free survival (PFS) and overall survival (OS) in stages II and III CC (7, 8). The benefit of adjuvant therapy in stage II CC is relatively small, and as such, it is not routinely administered (9). Patient preferences, treatment-related toxicities, and the risk characteristics of the tumor are considered in treatment decisions regarding adjuvant therapy.

Approximately 15% of colorectal cancers (CRCs) are dMMR/MSI-H, and patients with dMMR/MSI-H colon cancer are more likely to have a stage II disease (10). Mismatch repair (MMR) proteins are nuclear enzymes that bind to areas of abnormal DNA and repair base–base mismatch during cellular proliferation and division (11). Defects in DNA mismatch repair genes (MLH1, MSH2, MSH6, and PMS2) can lead to insertion or deletion of repeating nucleotide sequences in a process known as microsatellite instability (MSI). One third of these dMMR/MSI-H CC cases are inherited, known as Lynch syndrome carriers, and the rest are sporadic. MLH1 is considered the most commonly affected in the sporadic cases, which is more common in older patients and associated with BRAF V600E mutation (12). The microsatellite instability status of a tumor impacts the prognosis and benefit to adjuvant chemotherapy in patients with stage II CC (10–12). Multiple retrospective studies have shown that stage II patients with dMMR/MSI-H CC have a reduced metastatic potential and a more favorable prognosis compared to those with proficient mismatch repair (pMMR) tumors (13–16). In addition, previous retrospective studies of stages II and III colon cancer patients, analyzing data from randomized adjuvant therapy clinical trials, showed that stage II colon cancer patients with dMMR/MSI-H status did not benefit from adjuvant 5-FU-based chemotherapy (3, 10, 12, 17). Furthermore, Sargent et al. showed a decrease in overall survival (hazard ratio, 2.95; 95%CI, 1.02 to 8.54; *p* = 0.04) in dMMR/MSI-H stage II patients who were treated with single-agent 5FU compared to surgery alone (10). Whether high-risk features are prognostic in patients with dMMR/MSI-H stage II CC is not well established. The role of adjuvant chemotherapy in patients with high-risk dMMR/MSI-H stage II CC is not well defined. This study aimed to evaluate the prognostic value of high-risk features in dMMR/MSI-H stage II CC and their impact on adjuvant chemotherapy in high-risk stage II CC with dMMR/MSI-H.

## Materials and Methods

### Patient Selection

The National Cancer Database (NCDB) is a large cancer directory that represents approximately 70% of all newly diagnosed cancers in the US. The inclusion criteria for this study included the following: International Classification of Diseases for Oncology, third edition, morphological codes (8020, 8140, 8144, 8210, 8211, 8480, 8481, and 8490) and topography codes (C18.0-9), in participant user data files between the years 2010 and 2013. MSI status information was not available for patients diagnosed before 2010. The primary outcome was the overall survival of patients with dMMR/MSI-H stage II with high-risk features.

### Eligibility Criteria

Patient information was independently reviewed by two of the authors for the eligibility criteria. The patients were deemed eligible if they have dMMR/MSI-H stage II CC. Patients with mixed adeno-squamous histology, rectosigmoid location, and rectal cancer were excluded. Patients who received radiation therapy before or after surgery were excluded. Patients who received chemotherapy prior to surgery were excluded, as this may impact the pathologic stage at resection. High-risk features were defined as the following: <12 lymph nodes examined, lymphovascular invasion (LVI), positive surgical margin, pT4 tumor. No data were available for obstruction or perforation at diagnosis. Poor or undifferentiated histology was not included as a high-risk feature, as it is a good prognostic factor in dMMR/MSI-H stage II CC (18). High-risk stage II CC was defined as having at least one high-risk feature. Institutional approval and informed consent were not required for this study since the patient information in the database is completely de-identified, and the database is legally accessible to the public.

### Data Extraction and Statistical Analysis

The patient-specific covariates included were date of diagnosis, date of death, age, gender, race, tumor site, histology, insurance status, stage, presence of metastatic disease, co-morbid medical conditions, location of treatment, and treatment regimen (single or multi-agent chemotherapy). The treatment and clinical outcomes included overall survival rate. All data were checked for internal consistency.

### Analysis

All patients in the analysis had dMMR/MSI-H stage II colon cancer. Univariate and multivariate analyses were conducted to identify the factors associated with patient outcome (OS). The clinical and demographic characteristics of the patients were summarized using descriptive statistics as appropriate for variable type and distribution (chi-square test for categorical variables and ANOVA for numerical variables). Univariate and multivariate Cox-PH models were built to assess the association between patient characteristics and survival. Backward selection with an alpha level of removal of.05 was used in the multivariate analysis. The Kaplan–Meier survival curves were generated with log-rank tests to evaluate the association between adjuvant chemotherapy in high-risk and low-risk cohorts separately. All analyses were performed with a significance level of 0.05 (two-sided) with SAS Statistical Package, v9.4 (SAS institute, Inc., Cary, North Carolina).

## Results

**Figure 1:**
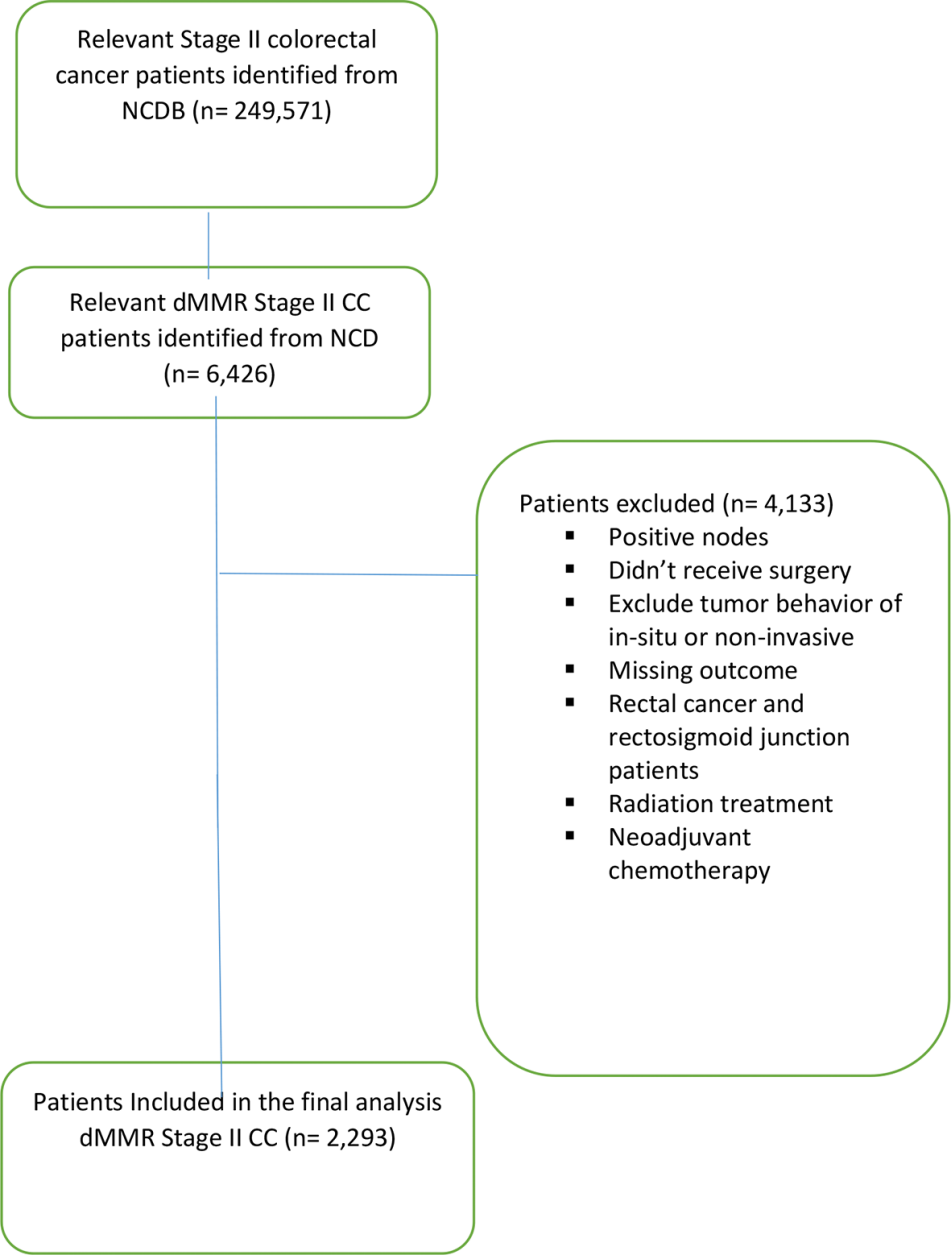
Consort diagram outlining the study selection.

Of the 249,571 patients with stage II colorectal cancer diagnosed between 2010 and 2013 in the NCDB database, 6,426 patients were determined to have dMMR/MSI-H status, and 2,293 met the inclusion criteria of the study (**Figure 1**). Females accounted for 58.2% of patients; 87.4% were Caucasian. The median age was 69 years (range, 21–90 years old). The most common tumor location was the ascending colon (32.5%), followed by the cecum (27.1%) and the transverse colon (13.1%). The sigmoid and the descending colon accounted for 9.7 and 5.0% of cases, respectively (**Table 1**).

**Table 1:** Demographics and clinical characteristics of patients.

Variable	*N* (%)
**Total**	2,293
**Gender**	
Male	958 (41.8)
Female	1,335 (58.2)
**Age**	
Median	69 (21–90)
STD	14.78
**Race**	
White	2,004 (87.4)
African American	188 (8.2)
Others	101 (4.4)
**Year of diagnosis**	
2010	413 (18.0)
2011	566 (24.7)
2012	633 (27.6)
2013	681 (29.7)
**Charlson–Deyo score**	
0	1,540 (67.2)
1	537 (23.4)
2+	216 (9.4)
**Site**	
Appendix	8 (0.4)
Cecum	621 (27.1)
Ascending colon	746 (32.5)
Hepatic flexure	143 (6.2)
Transverse colon	301 (13.1)
Splenic flexure	80 (3.5)
Descending colon	115 (5.0)
Sigmoid colon	222 (9.7)
Overlapping	36 (1.6)
Not otherwise specified	21 (0.9)
**Surgery at primary site**	
Partial colectomy	487 (21.2)
Subtotal colectomy/hemicolectomy	1,722 (75.1)
Total colectomy	42 (1.8)
Total proctocolectomy	10 (0.4)
Unknown	32 (1.4)
**Grade**	
Well differentiated/moderately differentiated	1,540 (67.2)
Poorly differentiated/undifferentiated	753 (32.8)
Pathological T stage	
T3	1,957 (85.3)
T4	321 (14.0)
**Risk group per study**	
High risk	676 (29.5)
Not high risk	1,617 (70.5)
High-risk features^a^	
T4	321 (14.0)
LVI	329 (14.3)
<12 lymph node removed	89 (3.9)
Positive margin	69 (3.0)
**Systemic/surgery sequence**	
No systemic therapy	1,862 (81.2)
Systemic therapy after surgery	431 (18.8)
**Chemotherapy**	
No chemotherapy	1,862 (81.2)
Single-agent chemotherapy	108 (4.7)
Multiagent chemotherapy	292 (12.7)
Chemotherapy type and number of agents not documented	31 (1.4)

a High-risk features not mutually exclusive.

In the entire cohort, 29.5% (*n* = 676) of patients were deemed to have a high-risk stage II CC. Positive margins, LVI, and less than 12 lymph nodes examined were observed as 3.0, 14.3, and 3.9%, respectively. pT4 was present in 14.0% of patients (**Table 1**). Of the high-risk patients, 36.1% (*n* = 244) received adjuvant chemotherapy, of whom 72.1% (*n* = 176) received multiagent therapy and 23.4% (*n* = 57) received single-agent chemotherapy, and 4.5% (*n* = 11) received an unknown number of agents.

On univariate analysis, high-risk status, pT4A/B tumor (pT3 as reference), pathological stage IIB/C (pathological stage IIA as reference), <12 lymph nodes (≥12 lymph nodes as reference), positive margins (negative margins as reference), Charlson–Deyo score >1 (0 as reference), and elder age (continuous scale) at diagnosis were associated with worse overall survival (**Table 2**). On multivariate analysis, male sex, positive surgical margin (negative margin as reference), Charlson–Deyo score >1 (0 as reference), high-risk disease, and older age at diagnosis were associated with worse OS (**Table 2**).

**Table 2:** Overall survival (OS) by mismatch repair and treatment status in univariate and multivariate analysis.

	Univariate OS		*p*-values	Multivariate OS		*p*-values
	HR	95%CI		HR	95%CI	
Age	1.05 (1.04–1.06)		<0.001	1.04 (1.03–1.06)		<.001
Male gender	1.02 (0.82–1.28)		0.843	1.36 (1.07–1.71)		0.004
High risk	1.56 (1.24–1.97)		<0.001	1.71 (1.35–2.17)		<0.001
Adjuvant chemotherapy	0.52 (0.36–0.73)		<0.001	0.66 (0.44–0.98)		0.040
Pathological T4A^a^	1.72 (1.19–2.49)		0.004			
Pathological T4B^a^	2.19 (1.53–3.14)		<0.001			
Pathologic stage IIB^a^	1.63 (1.13–2.35)		0.009			
Pathologic stage IIC^a^	2.27 (1.58–3.25)		<.001			
LVI^a^	1.34 (1.00–1.81)		0.248			
<12 LNS^a^	2.06 (1.35–3.15)		<.001			
Positive surgical margin^a^	2.79 (1.79–4.34)		<.001			
Charlson–Deyo score 1	1.71 (1.31–2.22)		<.001	1.45 (1.11–1.90)		<.001
Charlson–Deyo score 2+	3.89 (2.93–5.17)		<.001	2.72 (2.03–3.65)		<.001

a Removed from the multivariate model to avoid collinearity with high risk.

**Figure 2:**
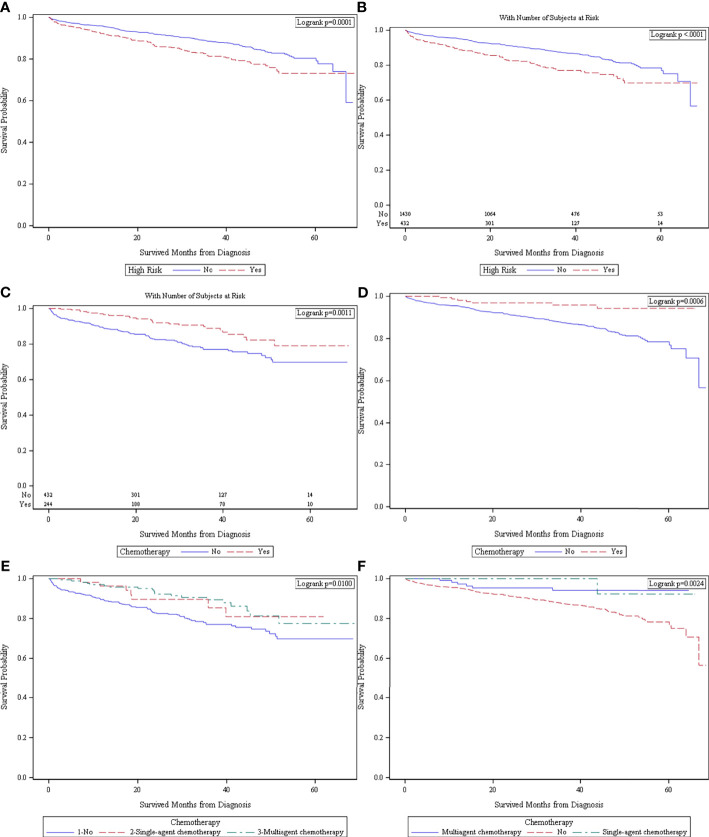
**(A)** Survival in high-risk (*n* = 676) *versus* no-high-risk (*n* = 1,617) patients in the entire cohort (*n* = 2,293) when not stratified by chemotherapy status. **(B)** Survival in patients who received no adjuvant chemotherapy (*n* = 1,862) in high-risk (*n* = 432) and no-high-risk (*n* = 1,430) patients. **(C)** Survival in high-risk patients (*n* = 676) who received adjuvant chemotherapy (*n* = 244) *versus* no adjuvant chemotherapy (*n* = 432). **(D)** Survival in patients with no high-risk features (*n* = 1,617) who received adjuvant chemotherapy (*n* = 187) *versus* no adjuvant chemotherapy (*n* = 1,430). **(E)** Survival in patients with high risk (*n* = 676) who received no adjuvant chemotherapy (*n* = 432), single-agent chemotherapy (*n* = 57), and multiagent chemotherapy (*n* = 176). **(F)** Survival in patients with no high-risk features who received no chemotherapy (*n* = 1,430), single-agent chemotherapy (*n* = 51), and multiagent chemotherapy (*n* = 116).

High-risk dMMR/MSI-H patients had worse OS compared to non-high-risk dMMR/MSI-H patients in the entire cohort when not stratified by status of adjuvant chemotherapy administration [5-year survival and 95%CI: 73.2% (67.3–78.1%) *vs*. 80.3% (76.7–83.5%), *p* = 0.0001, **Figure 2A**]. Median survival is not reachable in our cohort since none of the cohorts had more than 50% of patients who died at the end of follow-up; hence, 5-year survival was provided. In patients who received no adjuvant chemotherapy, the high-risk dMMR/MSI-H patients had worse OS [5-year survival and 95%CI: 69.8% (62.6–75.9%) *vs*. 78.4% (74.3–81.9%), *p* < 0.0001, **Figure 2B**].

High-risk dMMR/MSI-H patients who received adjuvant chemotherapy had better OS compared to those who had no chemotherapy [5-year survival and 95%CI: 78.0% (66.4–86.0%) *vs*. 69.8% (62.6–75.9%), *p* = 0.0011, **Figure 2C**]. In patients with no high-risk features, patients who received adjuvant chemotherapy had better OS [5-year survival and 95%CI: 94.3% (87.6–99.4%) *vs*. 78.4% (74.3–81.9%), *p* = 0.0006, **Figure 2D**]. In the patient groups by single/multi-agent adjuvant chemotherapy, single-agent and multi-agent chemotherapy patients demonstrated similar OS, which were both better than those with no chemotherapy. This finding is consistent in high-risk-feature (*p* = 0.01, **Figure 2E**) and no-high-risk feature patients (*p* = 0.0024, **Figure 2F**).

**Figure 3:**
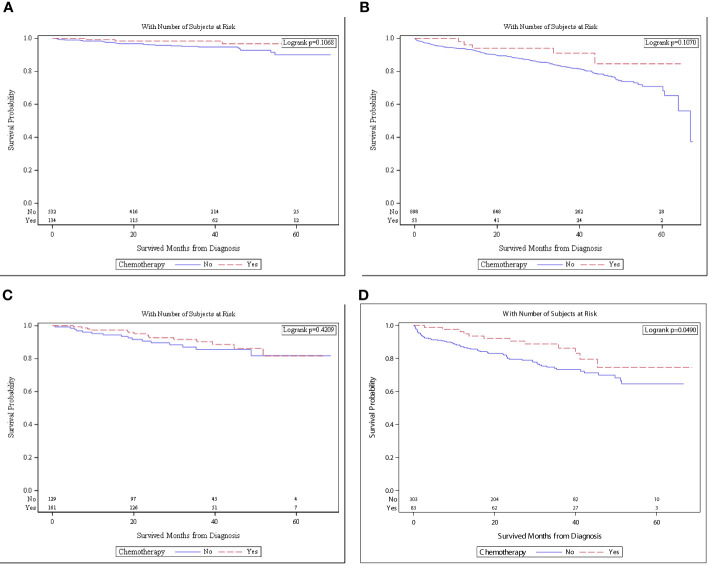
**(A)** Survival in patients with no high risk and <65 of age who received chemotherapy (*n* = 134) *versus* no chemotherapy (*n* = 532). **(B)** Survival in patients with no high risk and ≥65 of age who received chemotherapy (*n* = 53) *versus* no chemotherapy (*n* = 153). **(C)** Survival in patients with high risk and <65 of age who received chemotherapy (*n* = 161) *versus* no chemotherapy (*n* = 129). **(D)** Survival in patients with high risk and ≥65 of age who received chemotherapy (*n* = 83) *versus* no chemotherapy (*n* = 303).

When patients with no high-risk features were stratified by age, chemotherapy was no longer associated with better OS. The overall survival in patients with no high-risk features and aged <65 was not different with chemotherapy *versus* no chemotherapy [5-year OS and 95%CI: 96.7% (89.4–99.0%) *vs*. 90.0%: (83.9–93.9%), *p* = 0.1068, **Figure 3A**]. The overall survival in the same cohort aged ≥65 years was not different with chemotherapy *versus* with no chemotherapy [5-year survival and 95%CI: 96.1% (62.5–94.2%) and 70.9% (65.1–75.9%), *p* = 0.1070, **Figure 3B**].

The overall survival in patients with high-risk features was stratified by age. OS in patients aged <65 who received chemotherapy was not different than those of patients who did not receive chemotherapy [5-year OS and 95%CI: 81.7% (67.0–90.3%) *vs*. 81.8% (69.4–89.5%), *p* = 0.4209, **Figure 3C**]. The overall survival in the same cohort aged ≥65 years was superior with chemotherapy *versus* with no chemotherapy [5-year OS and 95%CI: 74.5% (56.2–86.1%) *vs*. 64.6% (55.5–72.3%), *p* = 0.0490, **Figure 3D**].

## Discussion

Prior reports and guidelines established the prognostic value of high-risk features in molecularly unspecified stage II CRC (9, 19–21). However, the therapeutic and prognostic implications of dMMR/MSI-H with high-risk clinicopathologic features have not been adequately studied. This study demonstrates that high-risk features are also prognostic in patients with stage II dMMR/MSI-H. This is the largest published study to establish the prognostic impact of high-risk features in dMMR/MSI-H stage II CRC.

The prognostic role of high-risk features in stage II dMMR/MSI-H setting raises the question regarding the role of adjuvant therapy in this group of patients. In this study, we observed a significant overall survival benefit in patients with high-risk dMMR/MSI-H stage II CC who received adjuvant chemotherapy compared with those who received surgery only. The benefit was associated with both multiagent and single-agent adjuvant chemotherapy. This is a novel finding that has a potential impact on clinical practice in the absence of data from clinical trials. The survival benefit persisted in patients >65 years when stratified by age. Sporadic dMMR/MSI-H is known to be associated with older age at diagnosis compared to germline dMMR/MSI-H (17, 22). In this study, patients with no high-risk features and dMMR/MSI-H stage II colon cancer also benefited from chemotherapy; however, age was an important confounding factor. Survival benefit was not evident in the low-risk group when stratified by age. We speculate that patients in the older age group of high-risk dMMR/MSI-H stage II CC of this study may have had a sporadic dMMR/MSI-H disease and may have derived more benefit from adjuvant chemotherapy compared to the younger population, which possibly may have had a higher rate of germline dMMR/MSI-H. It should be noted that no data were available in NCDB for BRAF status, MLH1 methylation status, and germline testing of family history.

Several prior studies, including retrospective analysis and observational reports, have attempted to address the role of adjuvant chemotherapy in stage II CC with high-risk features; however, these studies did not include the dMMR//MSI-H status (3, 23). Data about the role of adjuvant chemotherapy in high-risk dMMR/MSI-H stage II CC is limited. Tougeron et al. reported the clinical outcomes of stage II and III dMMR/MSI-H CC patients treated between 2000 and 2011 in a multicenter retrospective French study (24). Sixty percent (*n* = 149) of the patients were deemed to have high-risk factors, and 22% (*n* = 33) of the high-risk patients were treated with adjuvant chemotherapy. The high-risk features included pT4, VELIPI criteria (vascular emboli, lymphatic invasion, or perineural invasion), poor/undifferentiated histology, less than eight lymph nodes examined, tumor perforation, and initial bowel obstruction. Patients who were treated with adjuvant FOLFOX and not the single agent 5-FU showed a trend for longer disease-free survival compared to surgery alone. Although it included specific chemotherapy regimen information, the number of patients in that study was much smaller than in the present study.

Control for variables that could have influenced the results, such as histopathologic features, tumor grade, age, and performance status, was performed. Significant limitations still exist in this analysis, and these include retrospective design, lack of randomization, and no individualized patient data regarding the specifics of chemotherapy or follow-up. The reasons why adjuvant chemotherapy was not administered is unknown. The precise chemotherapy agents administered were not available. The sporadic *versus* germline mutational status is unknown, and the prevalence of other genomic alterations is similarly unknown. The overall survival is not cancer specific in this study, as NCDB includes only all-cause overall survival. In addition, the high-risk features did not include obstruction or perforation, as there was no data available in the database. Tumor perforation was shown to be associated with interperitoneal tumor dissemination (25), which raises the concern of whether these patients actually have stage II disease. The reported incidence of perforation and obstruction in stage II CC is less than 10% (26–28), and the impact of tumor cell spillage on recurrence and survival increases the risks substantially and may contribute to an increase in the benefit of adjuvant therapy.

## Conclusion

The prognostic value of high-risk features in dMMR/MSI-H stage II CC is confirmed. The prognostic value of high-risk features should be considered in adjuvant therapy discussions. Adjuvant chemotherapy may be associated with better OS in high-risk dMMR/MSI-H stage II CC patients, but significant limitations exist, including the retrospective nature of the data set. In the absence of randomized trials, the benefits and the risks of adjuvant therapy should be discussed with the patients with high-risk dMMR/MSI-H stage II CC. Further research needs to be done in the low-risk dMMR/MSI-H stage II CC to confirm the lack of benefit from adjuvant chemotherapy.

## Data Availability Statement

The original contributions presented in the study are included in the article/supplementary material. Further inquiries can be directed to the corresponding author.

## Author Contributions

AM and MA participated in collecting data, writing the manuscript, and editing the manuscript. MD participated in collecting data, writing the manuscript, and editing the manuscript. MB participated in the analysis of data and editing the manuscript. RJ, PP, CW, OA, WS, TG, and GB participated in editing the manuscript. BE-R participated in mentoring the whole project and editing the manuscript. All authors read and approved the final manuscript.

## Funding

The research reported in this publication was supported in part by the Winship Research Informatics Shared Resource of Winship Cancer Institute of Emory University and National Institutes of Health/National Cancer Institute under award number P30CA138292.

## Author Disclaimer

The American College of Surgeons and the Commission on Cancer have not verified and are not responsible for the analytic or statistical methodology employed or the conclusions drawn from these data by the investigator.

## Conflict of Interest

The authors declare that the research was conducted in the absence of any commercial or financial relationships that could be construed as a potential conflict of interest.

## Publisher’s Note

All claims expressed in this article are solely those of the authors and do not necessarily represent those of their affiliated organizations, or those of the publisher, the editors and the reviewers. Any product that may be evaluated in this article, or claim that may be made by its manufacturer, is not guaranteed or endorsed by the publisher.

## Abbreviations

dMMR, deficient mismatch repair; pMMR, proficient mismatch repair; MSI-H, microsatellite high; MSS, microsatellite stable.
